# Automated electronic health record-based screening for Fabry disease in unexplained left ventricular hypertrophy (FAPREV-HCM)

**DOI:** 10.1136/openhrt-2024-003116

**Published:** 2025-01-11

**Authors:** Kolja Lau, Victoria Sokalski, Lora Lorenz, Georg Fette, Claudia Sommer, Nurcan Üçeyler, Christoph Wanner, Peter Nordbeck

**Affiliations:** 1 Department of Internal Medicine I, Universitätsklinikum Würzburg, Würzburg, BY, Germany; 2 Fabry Center for Interdisciplinary Therapy (FAZiT), Universitätsklinikum Würzburg, Würzburg, BY, Germany; 3 Data Integration Center (DIZ), Service Center Medical Informatics (SMI), Universitätsklinikum Würzburg, Würzburg, BY, Germany; 4 Department of Neurology, Universitätsklinikum Würzburg, Würzburg, BY, Germany

**Keywords:** Cardiomyopathy, Hypertrophic, Genetic Diseases, Inborn, Metabolic Diseases, Epidemiology, Electronic Health Records

## Abstract

**Background and aims:**

Hypertrophic cardiomyopathy (HCM) has various aetiologies, including genetic conditions like Fabry disease (FD), a lysosomal storage disorder. FD prevalence in high-risk HCM populations ranges from 0.3% to 11.8%. Early diagnosis of FD is crucial due to available treatments, but its rarity and diverse symptoms complicate identification. Heart-specific FD variants often lead to late diagnoses due to the absence of typical FD symptoms. This prospective study (NCT04943991) was conducted to identify patients with undiagnosed FD using electronic health records (EHR) at a German tertiary-care hospital.

**Methods:**

Over 20 years (2000–2020), 2824 patients with ‘left ventricular hypertrophy (LVH)’ or ‘hypertrophic cardiomyopathy (HCM)’ were identified by full-text search. Exclusion criteria were age over 85, other diagnosed cardiomyopathies, significant valvular heart disease, death, active malignancy and prior FD testing. The remaining patients received an invitation for FD genetic testing.

**Results:**

Of the 2824 identified patients, 2626 (93%) fulfilled the exclusion criteria. Among the 198 included patients, 96 responded, and 55 underwent genetic testing, yielding a response rate of 48% and a testing rate of 28%. In one patient (1.8% of tested), FD was diagnosed with the *p.N215S* variant. Subsequent family screening revealed six additional FD cases, with four initiating FD-specific therapies. Comprehensive clinical evaluations were conducted in five of the seven identified patients.

**Conclusions:**

Genetic testing of patients with unexplained LVH/HCM using EHR is effective for identifying FD. Subsequent family screening further identified at-risk individuals, promoting regular follow-ups and if needed FD-specific therapies. This approach highlights the potential for broader application in high-risk populations to uncover treatable genetic conditions. The next phase should focus on automating the executed search process.

**Trial registration number:**

NCT04943991.

**WHAT IS ALREADY KNOWN ON THIS TOPIC**

- Fabry disease (FD) exhibits diverse phenotypes and variable severity, which, combined with its rarity, complicates diagnosis. This study aimed to investigate the prevalence of FD among patients who visited a large tertiary hospital in Germany with diagnosed left ventricular hypertrophy (LVH) or hypertrophic cardiomyopathy over a time span of 20 years, seeking to identify potentially undiagnosed cases using automated electronic patient record analysis.

**WHAT THIS STUDY ADDS**

- This study shows that automated electronic patient record analysis is feasible to identify so far undiagnosed patients with FD as the underlying aetiology of LVH, often followed by a relevant number of additional patients identified by subsequent family screening.

**HOW THIS STUDY MIGHT AFFECT RESEARCH, PRACTICE OR POLICY**

- Rare diseases often remain underdiagnosed due to challenges and hurdles in clinical routine. Our study outlines a novel approach to improve diagnostic rates for rare genetic diseases also feasible retrospectively, which appears increasingly important with new therapeutic options emerging.

## Background

 Hypertrophic cardiomyopathy (HCM) is caused by a variety of different aetiologies, ranging from inborn errors to acquired pathological conditions.^1^ Fabry disease (FD), a lysosomal storage disorder, is one of the genetic aetiologies of hypertrophic cardiomyopathy (HCM).^2^ The reported prevalence in high-risk HCM collectives ranges from 0.3% to 11.8%.^3^,^8^ With several therapeutic approaches available, FD is potentially treatable, making early diagnosis of the disease of paramount importance.^9 10^ However, due to the wide variety of symptoms, phenotypic expressions and rarity of the disease, early and effective diagnosis remains an obstacle.^11^ In particular, patients with heart-specific variants (eg, *p.N215S*) are often diagnosed late due to the lack of other more specific FD symptoms.^12^

## Aims

The aim of this prospective study was to investigate, whether an EHR-based algorithm can be used to identify so far undiagnosed FD patients in a large tertiary-care hospital in Germany.

## Methods

Over the past 20 years (2000–2020), patients visiting our hospital were screened using their EHR. Patient data was sampled using Patient DataWarehouse Navigator (PaDaWaN), a data query tool developed at University Hospital Würzburg, Germany.^13^ With this tool, we could perform a full-term search collecting cases fitting the term ‘left ventricular hypertrophy (LVH)’ or ‘hypertrophic cardiomyopathy (HCM)’.

Next, we evaluated the identified patients using straightforward exclusion criteria. Table 1 presents these criteria along with explanations for their application. Patients under 18 or over 85 years of age, as well as those with a pre-existing diagnosis of cardiomyopathy or tested FD, were excluded. Additionally, individuals with LVH due to an alternative explanation (eg, symptomatic valvular heart disease) were excluded. Patients undergoing active malignancy treatment were excluded for ethical reasons. Patient death was also an exclusion criterion. The remaining patients were included in the subsequent analysis. All participants received a mailed letter explaining FD, the importance of testing and potential therapeutic options and were invited to take part in our screening study. Interested patients were asked to visit our study centre for FD testing, which was conducted through genetic analysis of EDTA blood samples at CENTOGENE AG (Rostock, Germany). On receipt of the results, patients were informed of their test outcomes via phone. Those who tested positive were invited to undergo a comprehensive FD evaluation at our FD centre independently of the screening study.

**Table 1:** Exclusion criteria used for further analysis

Exclusion criteria	Explanation
Age<18 years or >85 years	FD-related LVH unlikely at this age. There are no known cases with LVH in patients with FD under 18 years. Undiagnosed patients over 85 years are not likely to have severe FD.
Genet. preconfirmed or excluded FD	Genetic testing report for FD was available in the EHR.
Preconfirmed other cardiomyopathy	Testing report for an alternative cardiomyopathy was available in the EHR (ie, sarcomeric mutations, Friedreich’s ataxia)
Symptomatic valvular heart disease	Reasonable explanation for LVH other than FD found.
Active malign disease	For ethical reasons patients with active malign disease under therapy were excluded. Patients with chemotherapy-associated cardiomyopathy were also excluded.
Death	Unable to retrospectively perform genetic testing.
Other reasons	Sufficient explanation for LVH (ie, certain metabolic diseases, severe long-standing secondary arterial hypertension, pheochromocytoma, morbid obesity, anabolic steroid abuse).

EHRelectronic health recordsFDFabry diseaseLVHleft ventricular hypertrophy

## Results

**Figure 1:**
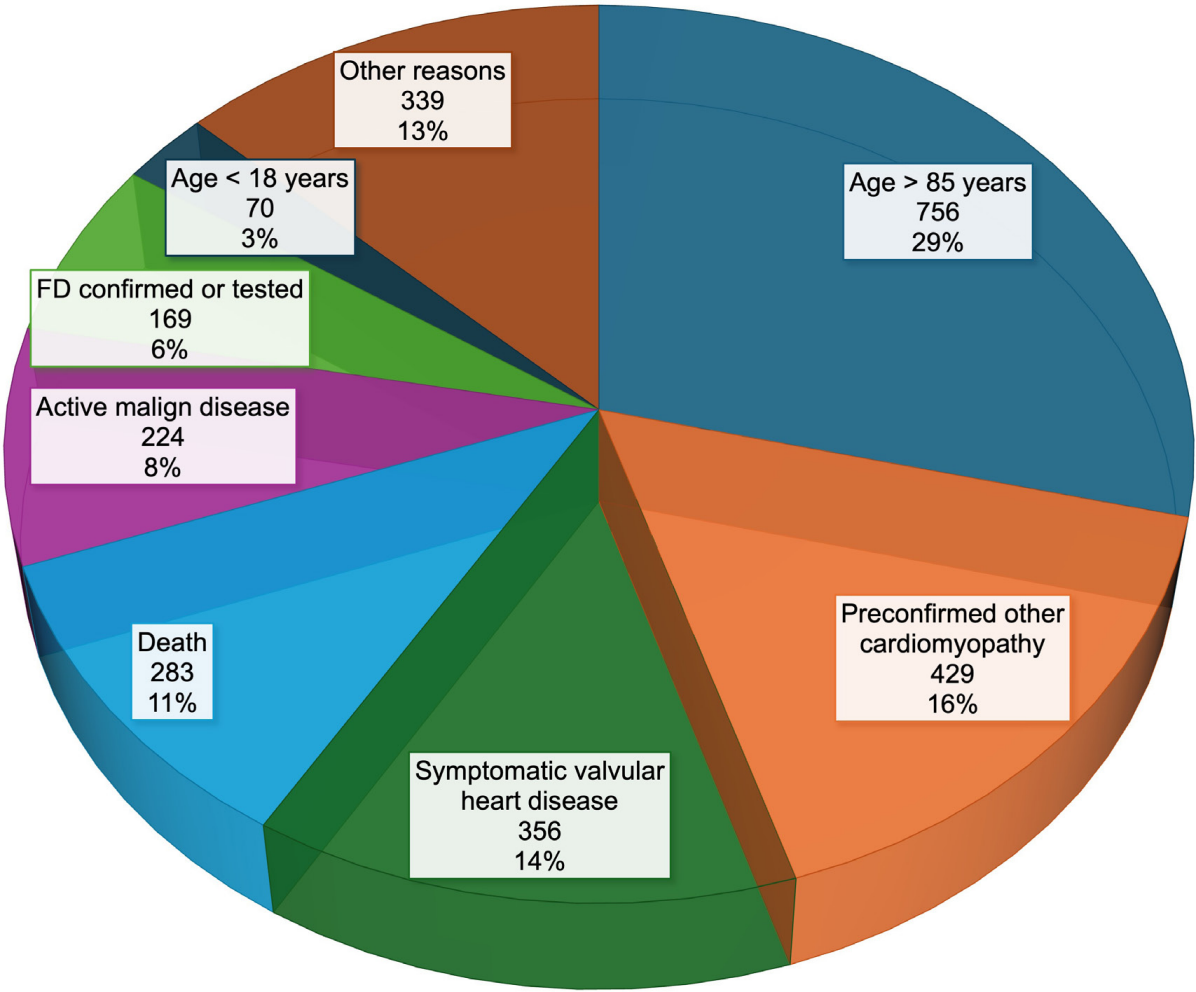
Exclusion criteria. Illustrates the exclusion criteria for the found patients with left ventricular hypertrophy. The primary reasons for exclusion were age over 85 years at the time of data analysis (29%), followed by a diagnosis of another cardiomyopathy (16%), and symptomatic valvular heart disease causing left ventricular hypertrophy (14%). FD, Fabry disease

The full-text search identified 2824 patients matching the term LVH/HCM. About 2626 (93%) patients fulfilled the exclusion criteria. The primary reasons for exclusion were age over 85 years (29%) at the time of data analysis, followed by a diagnosis of another cardiomyopathy (16%) and significant valvular heart disease causing the LVH (14%). Documented death (11%), active malign disease (8%), FD already tested or confirmed (6%) or age<18 years (3%) were also reasons for exclusion from further analysis. The remaining 13% were excluded for other reasons such as long-standing secondary arterial hypertension or other diagnosed metabolic diseases. Figure 1 illustrates the distribution of excluded patients.

**Figure 2:**
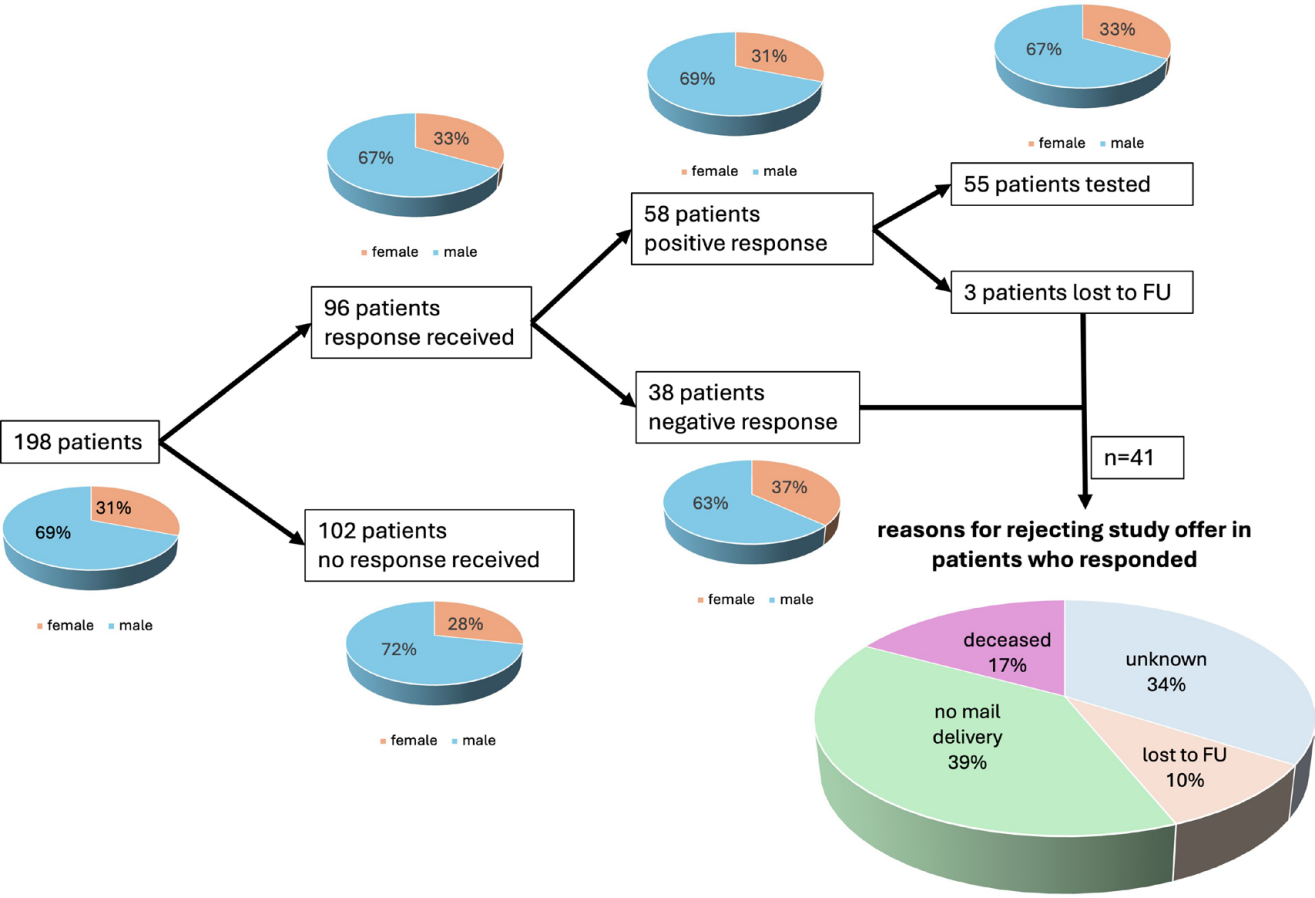
Details of included patients and reasons for rejecting the study offer. Provides details on the 198 patients (69% male) invited to participate in the study via letter. Of the initial 96 responses, 58 patients accepted the offer and 55 underwent FD testing. A negative response was received from 41 patients, with 39% unable to receive mail, 17% deceased, 34% offering no explanation and 10% lost to follow-up. FD, Fabry disease. FU, follow up.

Of the 198 (7%) included patients who were contacted by mail and offered a genetic testing for FD, 96 responded and 55 underwent genetic testing. This gave a response rate of 48% and a testing rate of 28%. Ultimately, one patient tested positive for FD (1.8% of tested patients). This patient was male and the genetic variant *p.N215S* was confirmed. Figure 2 provides an overview of the 198 patients (69% male) who received an invitation letter to participate in the study. Of these, 96 patients responded, with 58 agreeing to participate. Ultimately, 55 patients were tested for FD. No response was received from 102 patients. A negative response was received from 41 patients: 39% had undeliverable mail, 17% were deceased, 34% declined without specifying a reason and 10% were lost to follow-up.

**Figure 3:**
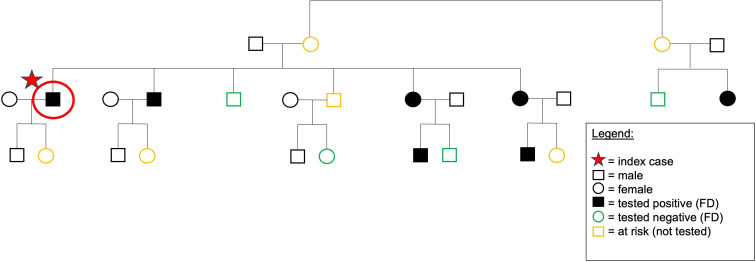
Family screening. Presents the results of family screening. In addition to the identified index patient, six family members were diagnosed with FD, four tested negative and six at-risk individuals were identified but have not yet been tested. FD, Fabry disease

Subsequent family screening identified an additional six individuals with FD (three males), while four related individuals tested negative, excluding a diagnosis of FD. Figure 3 depicts the pedigree and family screening results. All positively tested cases were referred to our centre for FD evaluation.

Five of the seven patients who tested positive underwent a comprehensive clinical evaluation. The median age was 50.8 years, with a gender distribution of four males and one female. Detailed results are provided in table 2. FD-specific therapy was initiated in four cases. All patients with positive test results were enrolled in annual or biennial follow-up protocols involving comprehensive clinical evaluations.

**Table 2:** Baseline characteristics of patients undergoing comprehensive post-diagnosis examination

	Male (n=4)	Female (n=1)	All patients (range)
Age (years)	47.5	63	34–63
A-Gal-enzyme activity (nmol/min/mg protein)Norm: 0.4–1.0 nmol/min/mg protein	0.05	0.32	0.04–0.32
Lyso-Gb3 (ng/mL)Norm: <0.9 ng/mL)	6.2	1.8	1.8–9.9
Nt-proBNP (pg/mL)	53	184	10–1487
Troponin (pg/mL)	6.9	8.3	5–22
GFR (mL/min/1.73 m^2^)	87.5	61	61–99
Albumin/creatinine (mg/g creatinine)	5.8	2	2–20
IVSD (mm)	10	7	7–18
LVPWD (mm)	8.5	7	7–10
Cardiac mass in MRI (g/m^2^)	80	44	44–130
LGE (n) (%)	2 (50)	1 (100)	
Therapy indication (%)	3 (75)	1 (100)	

Data is presented using the median, with ranges provided for all patients.

GFR, glomerular filtration rateIVSD, intraventricular septum thickness (measured in echocardiography); LGE, late gadolinium enhancement; LVPWD, left ventricular posterior wall thickness (measured in echocardiography)MRImagnetic resonance imaging

### Discussion

Subsequent genetic testing of patients with unexplained LVH or HCM stated in the EHR proved useful in identifying FD as the underlying aetiology. By defining simple exclusion criteria, one FD patient out of 55 referred to genetic testing was identified. Additionally, accessible family screening identified many at-risk individuals, leading to genetic testing, regular follow-ups and the initiation of FD-specific therapies in four patients.

In high-risk cohorts, large studies have reported prevalence rates between 0.3% (Kim *et al*, n=988 and 0.9% Azevedo *et al*, n=780).^7 14^ Our study, involving 198 patients, detected one positive case, resulting in a prevalence of 0.5%. The positive test rate among the 55 tested patients was 1.8%, consistent with predicted ranges.

The identified genetic variant *p.N215S* is associated with a distinct cardiac phenotype characterised by significant LVH without the manifestation of typical FD symptoms over time. Detection of this specific variant highlights the importance of performing FD testing in cases of equivocal LVH.

Initiating communication with high-risk patients resulted in a 48% response rate, indicating a notable interest in the proposed offer. Subsequently, 28% of respondents agreed to undergo FD testing. Several patients did not respond to our initial offer, likely due to long travel distances across Germany and limited communication options (mail only) imposed by data protection regulations. Negative responses included 39% due to undeliverable mail and 17% because the patient had already passed away (figure 2). These challenges reflect the extended inclusion period (2000–2020) and difficulties in patient contact, leading to a reduced response rate.

Our findings are constrained due to the lack of comprehensive testing of all patients identified in the full-text search. The extent to which FD diagnoses are missed remains uncertain, making it difficult to assess the effectiveness of our algorithm. For example, excluding all patients>85 years suggests that these patients cannot carry a significant FD variant. Similarly, excluding patients with severe aortic stenosis may also overlook the presence of an FD variant causing LVH. Further analysis should focus on evaluating the applied criteria also in excluded patients to assess their effectiveness and identify any missed FD cases.

Given the 1.8% positive test rate, the cost-effectiveness of our algorithm merits attention. Our results are consistent with expectations for a rare disease like FD. Through family screening, we identified six additional patients with FD at an early stage, facilitating timely treatment initiation and follow-up. These positive outcomes should be considered when interpreting our findings. Early disease detection can reduce costs associated with advanced disease stages.

Still, our study, despite the limitations mentioned above, identified 13 patients who benefited from the initial search or subsequent family screening. It demonstrated that electronic patient records can effectively identify individuals with rare diseases within high-risk groups. Further work should focus on automating this search through artificial intelligence and high-performance computing. Encrypted diagnosis within account records could furnish accessible data on at-risk patients across all healthcare settings. Rigorous genetic testing should be extended to high-risk individuals, especially where specific treatment is available.

## Data Availability

Data are available upon reasonable request.
